# Evaluation of the Lipophilicity of Angularly Condensed Diquino- and Quinonaphthothiazines as Potential Candidates for New Drugs

**DOI:** 10.3390/molecules29071683

**Published:** 2024-04-08

**Authors:** Daria Klimoszek, Małgorzata Jeleń, Beata Morak-Młodawska, Małgorzata Dołowy

**Affiliations:** 1 Faculty of Pharmaceutical Sciences in Sosnowiec, Doctoral School, Medical University of Silesia in Katowice, 40-007 Katowice, Poland; d201204@365.sum.edu.pl; 2 Department of Organic Chemistry, Faculty of Pharmaceutical Sciences in Sosnowiec, Medical University of Silesia in Katowice, Jagiellońska Street 4, 41-200 Sosnowiec, Poland; bmlodawska@sum.edu.pl; 3 Department of Analytical Chemistry, Faculty of Pharmaceutical Sciences in Sosnowiec, Medical University of Silesia in Katowice, 41-200 Sosnowiec, Poland; mdolowy@sum.edu.pl

**Keywords:** diquinothiazines, quinonaphthothiazines, lipophilicity, ADME, RP-TLC, phenothiazines

## Abstract

Lipophilicity is one of the most important properties of compounds required to estimate the absorption, distribution, and transport in biological systems, in addition to solubility, stability, and acid–base nature. It is crucial in predicting the ADME profile of bioactive compounds. The study assessed the usefulness of computational and chromatographic methods (thin-layer chromatography in a reversed-phase system, RP-TLC) for estimating the lipophilicity of 21 newly synthesized compounds belonging to diquinothiazines and quinonaphthiazines. In order to obtain reliable values of the relative lipophilicities of diquinothiazines and quinonaphthiazines, the partition coefficients obtained using different algorithms such as AlogPs, AClogP, AlogP, MLOGP, XLOGP2, XLOGP3, logP, and ClogP were compared with the chromatographic R_M0_ values of all the tested compounds measured by the experimental RP-TLC method (logP_TLC_). Additionally, logP_TLC_ values were also correlated with other descriptors, as well as the predicted ADME and drug safety profiling parameters. The linear correlations of logP_TLC_ values of the tested compounds with other calculated molecular descriptors such as molar refractivity, as well as ADME parameters (Caco-2 substrates, P-gp inhibitors, CYP2C19, and CYP3A4) generally show poor predictive power. Therefore, in silico ADME profiling can only be helpful at the initial step of designing these new candidates for drugs. The compliance of all discussed diquinothiazines and naphthoquinothiazines with the rules of Lipiński, Veber, and Egan suggests that the tested pentacyclic phenothiazine analogs have a chance to become therapeutic drugs, especially orally active drugs.

## 1. Introduction

Lipophilicity, defined as the partition coefficient between n-octanol and water (logP), has been an important descriptor for QSAR predictions for many years. The process of designing new drug substances typically covers a wide range of scientific disciplines, including microbiology, chemistry, and pharmacology. The search for new drugs begins with testing many new candidate molecules against a variety of biochemical targets. In addition to the biological activities of the drug candidates, parameters such as absorption and distribution in the human body are very important. The most popular route of drug transport is based on drug absorption through membranes by passive diffusion. Therefore, substances to be used as drugs must be lipophilic enough to penetrate the lipid core of membranes, but not too lipophilic to remain there [1,2,3,4,5,6]. Lipophilicity in drug design is an important factor influencing various chemical and biological properties of drugs. The lipophilicity of a drug affects its solubility in fats and other lipid-like substances. For many drugs to be effectively absorbed by the body, they must have appropriate solubility in lipids [7,8]. This parameter also plays a role in the ability of drugs to penetrate cell membranes. Molecules with higher lipophilicity often have an easier time crossing cell membranes, which can be crucial for drugs that need to reach the interior of cells to exert their effects [9,10]. Additionally, as documented in other papers, lipophilicity is determined using different separation techniques, including chromatography, which is a good predictor of blood–brain barrier penetration (BBB) of bioactive molecules. The BBB permeability plays a crucial role in the central nervous system (CNS) for potential drug candidates. Usually, a parabolic relationship between lipophilicity and drug entry into the brain is observed in vivo [11]. However, drugs with moderate lipophilicity often show the highest uptake. As was observed, high-polarity compounds exhibit high water solubility and fast clearance through the kidneys, and often contain functional groups (ionizable) that limit blood–brain barrier penetration [11]. However, more lipophilic compounds can be more vulnerable to P450 metabolism, leading to a faster clearance [11,12,13,14].

Many drugs act by interacting with specific proteins in the body. The lipophilicity of a drug can influence how strongly and selectively it binds to proteins, which in turn affects its biological activity. The lipophilicity of a drug may affect its metabolism in the body and the rate of its elimination. Molecules with higher lipophilicity may be more susceptible to accumulation in fatty tissues and remain in the body for longer [15]. With these factors in mind, when designing drugs, lipophilic properties should be taken into account, ensuring optimal solubility, penetration of cell membranes, and interactions with proteins, as well as metabolism and elimination. Therefore, research on the lipophilicity of drugs plays a crucial role in the process of developing new drugs.

The classic method for the direct experimental determination of lipophilicity is extraction in the n-octanol–water system. This method, called the shake flask method, is tedious and time-consuming, unsuitable for degradable compounds, and less amenable to automation. Therefore, chromatographic methods are becoming increasingly important in determining lipophilicity parameters. This method offers several practical advantages, including speed, repeatability, insensitivity to contaminants or degradation products, wider dynamic range, online detection, and reduced sample handling and sizes. In thin-layer chromatography and high-performance liquid chromatography, octadecylsilanized silica gel (RP-18) is used as the stationary phase, which, to some extent, imitates the structure of long-chain fatty acids in biological membranes. The mobile phase is usually a water–organic solution [16,17,18,19,20,21,22].

In addition to experimental methods for determining the logP parameter, computational methods are also very popular. These methods have several advantages over experimental methods, including short computation time and, more importantly, calculated logP parameters can be obtained before the synthesis. These facts mean that computational methods save time and chemical reagents, and are therefore very attractive from an economic and ecological point of view. The algorithms used to predict logP values are based on different theoretical methodologies: atomic approaches, piecewise contribution techniques, and property-dependent methods. However, as literature data show, the calculated lipophilicity is more or less similar to the experimental one. Depending on the algorithm used in the computational method and the structure of the tested substances, it may be a valuable complement to experimental methods, not a substitute [23,24,25,26,27].

Diquinothiazines and quinonaphthothiazine are modified pentacyclic phenothiazine structures into which two quinoline or quinoline and naphtalene rings have been introduced instead of two benzene rings. Such a modification allows for obtaining various isomeric diquinothiazines and quinonaphtothiazines that differ in the ring fusion method. So far, linearly, one-side-angularly- and double-angularly-fused diquinothiazines have been described in the literature. N-substituted diquinothiazines and quinonaphtothiazines exhibit strong antioxidant action and a significant effect against tens of cancer cells derived from leukemia, colon, ovarian, CNS, melanoma, renal, prostate, skin, breast, and non-small cell lung cancers, whereas NH-diquinothiazines show strong antioxidant activity. The most promising diquinothiazine, 6-chloro-ethylureidoethyldiquinothiazine, exerted suppressive and anti-inflammatory activities in the above-mentioned in vivo models, and showed inhibitory activity of IFNβ expression and IFNβ-dependent downstream genes and proteins involved in the pathogenesis of autoimmune diseases [28,29,30,31,32]. Dichinothiazines **1**, **9,** and **11** showed strong free radical scavenging, lipid peroxidation, and ion chelating effects. Compounds **1**, **9**, and **11** also inhibited alpha-glucosidase and alpha-amylase activities by reducing oxidative stress. In silico docking of these compounds shows a strong interaction with aldose reductase, glyoxalase 1, receptor AGE, alpha-glucosidase, and alpha-amylase [32].

The purpose of this work is to determine the following lipophilicity parameters: logP_calcd_, R_M0_, and logP_TLC_ of 21 anticancer and antioxidant one-side-angularly-condensed diquinothiazines **1**–**13** and naphtoquinothiazines **14**–**21** (Table 1) using computational programs and the RP-TLC method. Additionally, it is to discuss the influence of the nature of substituents and the method of ring condensation in a five-ring molecule system, and compare calculated data with experimental data; as well as analyze molecular descriptors, and ADME properties.

## 2. Results and Discussion

Lipophilicity studies were carried out on a series of previously synthesized pentacyclic, angularly condensed diquinothiazines (**1**, **5**, **9**, **11**) and quinonaphthothiazines (**14** and **18**) tested for selected biological activities, and their methyl (**2**, **6**, **10**, **12**, **15** and **19**), allyl (**3**, **7**, **13**, **16**, **20**), and propargyl (**4**, **8**, **17** and **21**) derivatives. During the first stage of the research, eight most-popular computer programs based on different algorithms were used. The calculated logP_calcd_ values for the tested compounds (**1**–**21**) differed depending on the substituents on the thiazine nitrogen atom and whether the structure contained two quinoline rings or one quinoline and one naphthalene system, as well as the program used. Most of the programs used in the research did not provide different values for individual isomers. The AClogP, ALOGP, XLOGP2, and XLOGP3 programs calculated the same values of the logP_calcd_ parameters for all isomeric diquinothiazines, **1**, **5**, **9,** and **11**, also for all their methyl, allyl, and propargyl tested derivatives. In the remaining four programs used, only some isomers were distinguished, e.g., the ALOGPS program calculated the value of the logP_calcd_ parameter for diquinotiazines **1** and **9** as 4.90 and 4.92, respectively, while for diquinotiazines **5** and **11,** it was 4.93. The MLOGP program allowed for obtaining the value of logP_calcd_ = 2.78 for derivative **1**, while for the remaining three structural isomers, the logP_calcd_ was 3.17. Performing calculations with the same programs for isomeric angularly condensed quinonaphthothiazines, the same results were obtained for pairs of isomers with the same substituents. The logP_calcd_ values varied significantly from 2.78 to 6.84 (Table 2). The highest lipophilic values were found for 7-methylquinonaphthothiazine **16** and 14-allylquinonaphthothiazine **20** (AclogP = 6.84). The lowest lipophilic values were found for 7*H*-diquino[3,2-b;3′,4′-e]thiazine **1** (MLOGP = 2.78). For all the tested diquinothiazines and quinonaphtothiazines (**1**–**21**), the lowest lipophilic value was found using MLOGP, while the highest using ClogP and AclogP. For the same compounds, the calculating programs gave various values, varying up to 2.59 in the logarithmic scale.

During a further stage of this research, in order to obtain reliable values, the relative lipophilicities of diquinothiazines and quinonaphtothiazines **1**–**21** expressed by the chromatographic values of R_M0_ were measured by the experimental RP-TLC method. The obtained R_M_ values decreased linearly with the increasing concentration of acetone in the mobile phase used. Table 3 shows the values of R_M0_ (intercept), b (slope), r (correlation coefficient), and logP_TLC_. The R_M0_ parameter describes the partition of examined compounds into the nonpolar stationary and polar mobile phases. The values are in the range of 3.15–4.60. The least lipophilic were compounds containing hydrogen at the nitrogen thiazine atom (**1**, **5**, **9**, **11**, **14**, **18**). In the group of six tested unsubstituted derivatives (NH), structures containing a naphthylene ring—quinonaphthothiasines (**14**, **18**)—turned out to be more lipophilic than diquinothiazines. Analyzing individual unsubstituted derivatives and their methyl, allyl, and propargyl derivatives, it can be seen that for each series of derivatives, thiazines containing an allyl substituent (**3**, **7**, **13**, **16** and **20**) turned out to be the most lipophilic.

As the R_M0_ values showed relative lipophilic properties of diquinothiazines and naphtoquinothiazines **1**–**21**, it was of interest to estimate the absolute lipophilic properties as logP values. The obtained R_M0_ values were recalculated using a calibration curve determined under the same measurement conditions for a set of standards **I**–**V,** with the literature values of logP_lit_ in the range of 1.21–6.38 (Table 4). The correlation between the logP_lit_ values and the experimental R_M0_ values for standards **I**–**V** gave the following calibration equation:

log P_TLC_ = 1.2838 R_M0_ + 0.2138 (r = 0.997; s = 0.192; F = 459.32; *p* < 0.001)

As can be observed, the obtained logP_TLC_ values were in the range of 4.26–6.15 (Table 3). The most lipophilic compound is naphthoquinothiazine **16** with an allyl substituent at the nitrogen atom (logP_TLC_ = 6.15), but the least lipophilic properties were found for derivative **1**, i.e., diquinothiazine unsubstituted at the thiazine nitrogen atom (logP_TLC_ = 4.26). Analyzing the values of lipophilicity parameters obtained experimentally, the lowest values of the logP_TLC_ parameter within individual isomers were obtained for thiazines unsubstituted at the thiazine nitrogen atom, while the highest for thiazines with allyl substituents. Also, the experimental values of the logP_TLC_ parameter turned out to be higher for naphthoquinothiazines than for diquinothiazines.

Next, the obtained experimental logP_TLC_ values of all studied compounds were compared with the calculated values (logP_calcd_) using cluster analysis.

**Figure 1:**
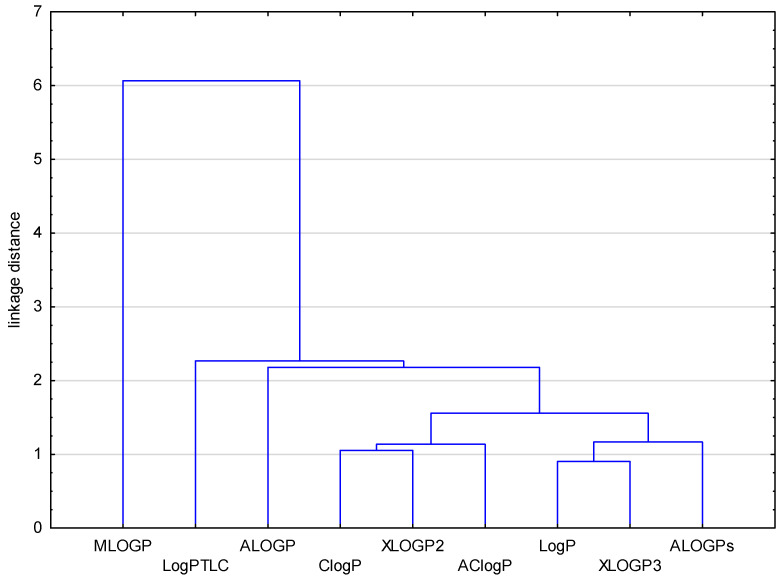
Dendrogram of similarity of chromatographical and theoretical values of the lipophilicity for the investigated compounds (**1**–**21**).

The cluster (similarity analysis) was carried out for all data obtained, taking into consideration both the experimental (logP_TLC_) and theoretical (AlogPs, AclogP, AlogP, MlogP, XlogP2, XlogP3, LogP, ClogP) values of lipophilicity. The results of this analysis are shown in Figure 1.

As can be seen in Figure 1, all theoretically obtained partition coefficients for the examined compounds (**1**–**21**) indicate a certain similarity, and form one large cluster, except for the MlogP parameter which has the highest distance on the presented dendrogram. The smallest Euclidean distance, and thus the biggest similarity, was present ClogP with XLOGP2 and AClogP, as well as LogP with XLOGP3 and ALOGPs. The experimental, i.e., chromatographically, determined parameter of lipophilicity as LogP_TLC_ is similar to ALOGP and next to the other above-mentioned LogP values. This analysis confirms the difference in the predictive power of applied calculation programs.

**Figure 2:**
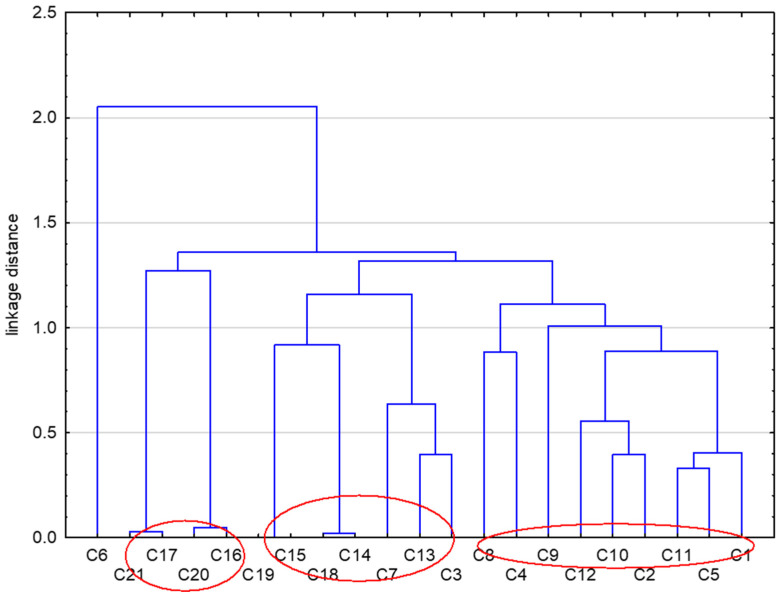
Dendrogram of similarity of the tested compounds (**1**–**21**) obtained on the basis of their lipophilicity parameters.

In further steps of this study, we applied the similarity analysis to compare the studied compounds based on all parameter lipophilicity obtained for them (Figure 2).

The data presented in Figure 2 indicate that the studied compounds can be divided into three groups taking into account both their chromatographic and theoretical lipophilicity parameters. The first subset consists of compounds **16**, **17**, **20**, and **21**, and the second **3**, **7**, **13**, **14**, **15**, **18**, and **19**. The third, and the biggest, cluster consists of compounds **1**, **2**, **4**, **5**, **8**, **9**, **10**, **11**, and **12**.

It can be therefore suggested that the applied cluster analysis can be a good tool for grouping these new molecules based on their lipophilicity.

Next, Table 5 shows the linear correlations obtained between the calculated partition coefficients, such as AClogP, MLOGP, XLOGP2, XLOGP3, ClogP, and LogP with experimental, i.e., chromatographic parameters of lipophilicity of these compounds, denoted as logP_TLC_.

It can be observed that the best correlation with coefficient r = 0.670 (Table S1) was obtained for AClogP values. The weakest correlation presented in Table S1 is demonstrated by ClogP at r = 0.450. Among all the calculated partition coefficients, unsatisfactory correlation with coefficient r = 0.295–0.359 was obtained for AlogP and AlogPs, respectively. It seems that the lipophilicity of compounds is not the only parameter determining biological activities because the prediction methods do not always include all structure aspects. The greatest inconvenience in using the tested computer programs to calculate lipophilicity parameters seems to be the fact that they show no difference for isomeric compounds. When determining lipophilicity experimentally for all isomeric diquinothiazines and naphthoquinothiazines, different values of R_M0_ parameters, and therefore logP_TLC_, were observed.

Later in the work, in order to assess the usefulness of the lipophilicity parameters determined experimentally, i.e., by the RP-TLC method, for predicting other structural descriptors of the tested compounds and their ADME parameters listed in Table S2 and Table 5, respectively, the previously obtained logP_TLC_ values were correlated with these descriptors. On the other hand, Table S3 demonstrates the obtained linear correlations between a proper molecular descriptor, such as molar mass and molar refractivity, as well as other ADME activities with the lipophilicity parameter logP_TLC_ of the studied compounds.

It was found that the logP_TLC_ values for all the investigated compounds (**1**–**21**) were correlated with molecular descriptors such as molar volume (V_M_) and molar refractivity (Ref_M_) (Table S2), which found weak-to-moderate results r = 0.428–0.445 and *p* = 0.053 and 0.043, respectively (Table S3). Weak correlation results from the fact that the obtained values of the molecular descriptor parameters have the same values for all isomeric diquinothiazines and naphthoquinothiazines, while the experimental logP_TLC_ parameter values are different for each isomer.

The logP_TLC_ values were also correlated with a predicted ADME and drug safety profiling parameters such as the Caco-2, PPB, CNS, HIA, P-gp substrates, CYP1A2 inhibitor, CYP2C9 inhibitor, CYP2C19 inhibitor, CYP2D6 inhibitor, and CYP3A4 inhibitor. The parameters discussed were obtained using the Percepta program from ACD labs [34], which predicts the pharmacokinetic properties of small molecules. The pharmacokinetic results of the compounds are presented in Table 5.

This method, like other calculation methods intended for predicting various pharmacokinetic parameters as well as directions of the activity of substances with potential biological activity, is subject to the risk of obtaining results that do not fully match experimental data. However, due to the high costs and time-consuming nature of performing these experimental tests in new drug design, it was considered acceptable to perform in silico tests at the first stages of searching for biologically active substances among newly synthesized angularly condensed diquino- and quinonaphthothiazines. Since lipophilicity parameters are one of the many factors influencing ADME parameters, we also attempted to correlate the logP_TLC_ parameter with selected ADME descriptors. Table S3 shows these correlations. As can be seen in Table S3, the obtained correlations are not satisfactory, which shows that in silico ADME profiling can provide only initial information about the pharmacokinetic properties of studied drugs. Therefore, in the future, for clinical trials, the critical review of theoretically obtained ADME properties of the analyzed compounds and obtained correlation equations and their comparison with experimental data is needed.

The most frequently used in vitro method for determining absorption orally administered drugs is to test the permeability of compounds through the Caco-2 cell monolayer. This is due to the functional and morphological similarity of Caco-2 cells to human intestinal epithelial cells [39].

The predicted Caco-2 permeability for the tested compounds is given as the Pe parameter in 10^−6^ cm/s. Compounds having a predicted Pe in 10^−6^ cm/s greater than 7.0 are considered to have high Caco-2 permeability. According to the results of the analysis, all the synthesized compounds showed a high permeability of Caco-2 cells (values ranged from 94 to 235).

Drugs predominantly attach to plasma proteins, with only a small percentage remaining unbound. Only the free and unbound fraction of the compound in the bloodstream can diffuse, be transported across biological membranes, or interact with receptors and enzymes. These drug–protein complexes act as reservoirs for the drugs. Therefore, in determining the ADME profile of the tested molecule, it is important to determine this parameter. All the investigated compounds exhibit a high PPB (PPB = 99–100%). The least PPB values were found for diquinothiazines **1**, **2**, **5**, **6**, and **9**–**12** (99%). For the remaining diquinothiazines and all tested quinonaphthothiazines, the value of the PPB parameter was calculated as 100% and is defined as extensively bound.

Calculated human intestinal absorption values (HIA) showed that all investigated diquinothiazines and quinonaphtothiazines (**1**–**21**) had an excellent probability of intestinal absorption (HIA = 100, Table 5).

P-glycoprotein, also called multidrug-resistant protein 1—MDR-1, is a membrane transporter belonging to the ABC superfamily of proteins (ATP-binding cassette). This is one of the most important membrane transporters, of which the primary function is the elimination of many substances from cells, both endogenous and exogenous. This protein also co-creates tissue barriers. For this reason, P-glycoprotein determines the bioavailability of many drugs with very different structures. Chemical and drug interactions with this protein may cause its changes in the bioavailability in situations where they are administered simultaneously. A wide spectrum of substances that are P-gp substrates includes metabolic products, lipids, sterols, and xenobiotics. The last group includes pharmacologically diverse drugs (anti-inflammatory, antiviral, anticancer, antibiotics, antidepressants). Most of the substances transported by P-glycoprotein are hydrophobic [40,41].

The calculation results for the tested substances (**1**–**21**) show that all unsubstituted diquinothiazines and quinonaphthothiazines will be not constitute substrates for P-gp (score < 0.3). However, the values obtained for the remaining substances are in the undefined range (score in the range of 0.33 to 0.67).

The metabolism of the investigated diquinothiazines and quinonaphtothiazines **1**–**21** was assessed in terms of their inhibitory effect on the main cytochrome P450 enzymes: CYP1A2, CYP2C9, CYP2C19, CYP2D6, and CYP3A4. Literature data show that these enzymes are responsible for the metabolism of nearly 80% of drugs [42,43].

The inhibition and induction of CYPs are major mechanisms causing pharmacokinetic drug–drug interactions, affecting the biotransformation and clearance of the drug, and may lead to an increase in its plasma concentration. CYP inhibition therefore leads to increased toxicity or a lack of therapeutic effect of the drug [44].

CYP1A2 is one of the major CYPs in the human liver and metabolizes many important drugs, such as clozapine, lidocaine, theophylline, tacrine, and leflunomide. CYP1A2 is one of the main enzymes bioactivating many procarcinogenic agents; therefore, CYP1A2 induction may increase the carcinogenicity of these compounds. This enzyme also metabolizes several important endogenous compounds, including steroids, retinols, melatonin, uroporphyrinogen, and arachidonic acid. CYP2C9 is mainly responsible for the metabolism of drugs with a narrow therapeutic index. CYP2C19 metabolizes several drugs (omeprazole, citalopram, voriconazole, cyclophosphamide) and is involved in cholesterol metabolism and steroid hormone metabolism. CYP2D6 is highly polymorphic and is responsible for the metabolism of 20% of the drugs. Some people may have an increased or decreased activity of this enzyme, which may affect the side effects and reduce the effectiveness of the drug. Cytochrome CYP3A4 is highly expressed in the human liver and small intestine, it is an enzyme that plays a major role in the metabolism of a wide variety of xenobiotics, including approximately 50% of therapeutic drugs, as well as many endogenous compounds [45,46,47,48,49,50].

Calculations regarding the inhibition of CYP enzymes show that derivatives **4**, **8**, **17**, and **21** containing a propargyl substituent and **15** and **19** with a methyl substituent at the thiazine nitrogen atom may be CYP1A2 inhibitors. For most of the diquinothiazines and quinonaphthothiazines tested, no CYP2C9 inhibition was predicted, while the inhibition scores of the remaining three CYP enzymes were in the undefined range.

To confirm the bioavailability of the tested compounds (**1**–**21**), they were checked for compliance with Lipiński’s, Ghose’s, Veber’s, and Egan’s rules (Table S4). For this purpose, the SwissADME platform was used [51]. The assessment of the usefulness of a substance as an orally administered drug based on its properties is defined by Lipinski’s rule of five [52,53]. Lipiński’s rule assumes that for good bioavailability, a substance should have a molecular weight < 500, no more than five hydrogen bond donors, no more than ten hydrogen bond acceptors and a partition coefficient value logP < 5. A substance is considered to meet this rule if it exhibits three of these parameters. When using a Ghose rule, substances should fulfill the following requirements: molar mas of 160 to 480 g/mol, logP of 0.4 to 5.6, atom count of 20 to 70, and refractivity of 40 to 130 [54]. However, according to Veber’s rule, for good bioavailability, a substance should have at most 10 rotatable bonds and a TPSA of at most 140 Å [55]. To determine whether a substance is well or poorly absorbed, Egan built a statistical model for recognizing patterns of passive intestinal absorption based on PSA and AlogP parameters. These descriptors are quite simple to interpret, which increases the usefulness of the model. It has been shown that the molecular weight, although often used in passive absorption models, is redundant because it is already a component of both the PSA and AlogP [56]. All discussed diquinothiazines **1**–**14** and naphthoquinothiazines **15**–**21** meet Lipiński’s, Veber’s, and Egan’s rules. Ghos’s rule is only unfulfilled in the case of naphthoquinothiazines **16** and **20** with an allyl substituent. These results indicate that the tested pentacyclic phenothiazine analogs have a chance of becoming drugs, especially orally active drugs.

## 3. Materials and Methods

### 3.1. Materials

In the experimental studies, the following reagents were used to prepare the mobile phase: acetone (POCh, Gliwice, Poland), TRIS (tris (hydroxymethyl) aminomethane, and Fluka. Five chemical compounds with the described lipophilicity parameter (logP_lit_) were used to prepare the calibration curve acetanilide (I, 1.21 [36]), benzoic acid (II, 1.87 [37]), benzophenone (III, 3.18 [37]), anthracene (IV, 4.45, [37], and p,p’-DDT (1,1,1-trichloro-2,2-bis(4-chlorophenyl), V, 6.38, [38]). In the previously described reactions, diquinothiazines and quinonaphtothiazines **1**–**21** were obtained [29,31].

### 3.2. Chromatographic Procedure

RP-TLC was carried out on a silica gel 60 RP-18 F_254S_ 10 cm × 10 cm RP-TLC plate (Merck, Darmstad, Germany). The mobile phase was acetone and an aqueous TRIS (tris(hydroxymethyl)-aminomethane) buffer pH = 7.4 (ionic strength 0.2 M) to meet physiological conditions. The concentration of acetone in the mobile phase ranged from 50 to 85% (*v*/*v*) in 5% increments. Angularly condensed diquino- and quinonaphthothiazinethiazines (1–21) and the standards I–V were dissolved in ethanol (2.0 mg/mL). Solutions (2 µL) of the analyzed compounds were applied to the plates 5 mm apart and 10 mm from the lower edge and sides of the plates. Before developing the plates, the chromatographic chambers were saturated with the mobile phase for 0.5 h. After developing the plates and drying in a stream of air, the chromatograms were observed under UV light at λ = 254 nm. At least, three chromatograms were developed for each soluble–solvent combination, and the R_F_ values were averaged. The R_M_ values calculated from the experimental R_F_ values using the equation R_M_ = log (1/R_F_-1) were linearly dependent on the concentration of acetone. The R_M0_ values were obtained by extrapolation to a zero acetone concentration using the equation R_M_ = R_M0_ + bC, where C is the concentration (%, *v*/*v*) of acetone in the mobile phase.

### 3.3. Computational Programs

The computer programs VCCLAB [33], Percepta [34], and ChemDraw [35] were successfully applied to predict the theoretical values of partition coefficients of the studied compounds in the form of AlogPs, AClogP, AlogP, MLOGP, XLOGP2, XLOGP3, logP, and ClogP. Statistica 13.3 software was used to perform the cluster analysis of these parameters. The calculations were performed on Euclidean distances and single linkage distances [57].

## 4. Conclusions

The aim of this study was to assess the lipophilicity of newly synthesized compounds from groups of diquinothiazines and quinonaphtothiazines. In order to obtain reliable values of the relative lipophilicities of diquinothiazines and quinonaphtothiazines, the partition coefficients obtained using various algorithms such as AlogPs, AClogP, AlogP, MlogP, XLOGP2, XLOGP3, logP, and ClogP, they were compared with the chromatographic values of this parameter of all studied compounds measured by the experimental RP-TLC method (logP_TLC_). Comparison of theoretical and chromatographic lipophilicity parameters, i.e., logP_calcd_ and logP_TLC_, for the tested compounds shows some differences in values. It was observed that theoretical, i.e., logP values calculated using algorithms, do not allow for distinguishing the isomeric forms of the tested compounds. On the other hand, for isomeric diquinothiazines and naphthoquinothiazines, different logP_TLC_ parameter values were obtained. Using computational and experimental techniques, the same relationship was obtained as that of the mutual parameter values within the individual subgroups of the tests; in the case of each thiazine group tested, thiazines unsubstituted at the nitrogen atom of thiazines were characterized by lower lipophilicity, and those with allyl substitutes were the most lipophilic. A similar relationship was also obtained in the lipophilicity of diquinothiazine–naphthoquinothiazine. In both methods used, higher values of the logP parameter were obtained for naphthoquinothiazines than for diquinothiazines. Linear correlations of logP_TLC_ values of the tested compounds with other calculated molecular descriptors, such as molar refractivity, as well as ADME parameters (Caco-2 substrates, P-gp inhibitors, CYP2C19, and CYP3A4) generally show poor predictive power. Therefore, in silico ADME profiling can only be helpful at the initial step of designing these new candidates for drugs. Weak correlations of the logP_TLC_ lipophilicity parameters with the described pharmacokinetic parameters result from the fact that the lipophilicity parameters are important, but not the only, factors influencing the pharmacokinetics of the substance. However, the prediction of pharmacokinetic properties at an early stage evaluation of candidates for new drugs allows for reducing the number of compounds intended for further research, while attempts to correlate these parameters with experimentally determined lipophilicity parameters may guide further experimental research. For further studies, it is planned to compare the obtained ADME parameters with experimental data for the tested compounds. The compliance of all discussed diquinothiazines and naphthoquinothiazines with the rules of Lipiński, Veber, and Egan suggests that the tested pentacyclic phenothiazine analogs have a chance to become therapeutic drugs, especially orally active drugs.

## Figures and Tables

**Table 1:** Structure of angularly condensed diquinothiazines and quinonaphtothiazines (**1**–**21**).

No. of Compound	Chemical Structure	No. of Compound	Chemical Structure	No. of Compound	Chemical Structure
**1**	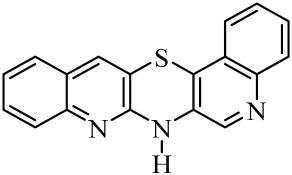	**8**	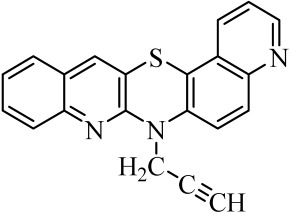	**15**	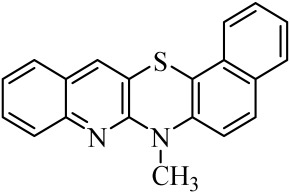
**2**	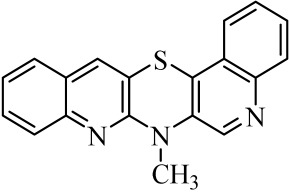	**9**	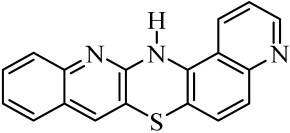	**16**	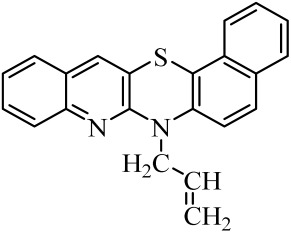
**3**	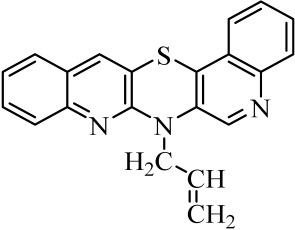	**10**	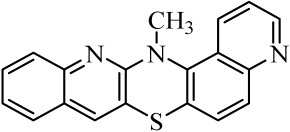	**17**	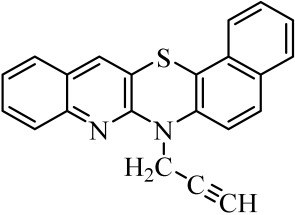
**4**	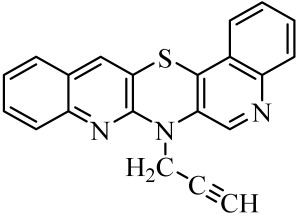	**11**	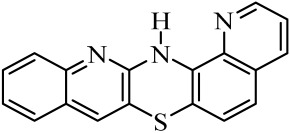	**18**	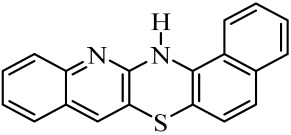
**5**	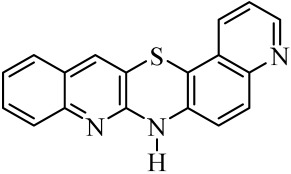	**12**	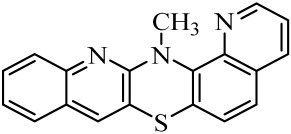	**19**	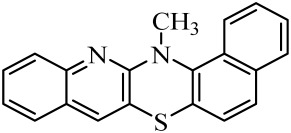
**6**	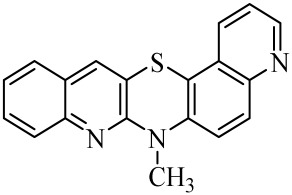	**13**	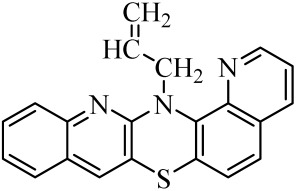	**20**	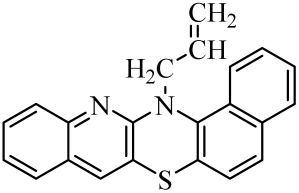
**7**	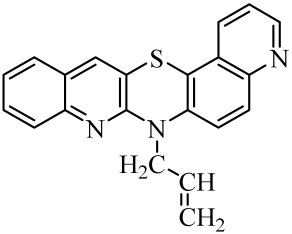	**14**	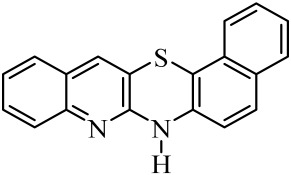	**21**	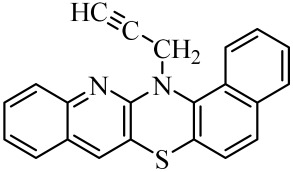

**Table 2:** The calculated lipophilic parameters (logP_calcd_) for angularly condensed diquino- and quinonaphthothiazines (**1**–**21**) using the internet data bases VCCLAB [33] and Percepta * [34] and ChemOffice ** [35].

No. of Compound	ALOGPs	AClogP	ALOGP	MLOGP	XLOGP2	XLOGP3	LogP *	ClogP **
**1**	4.90	4.66	4.58	2.78	4.72	4.48	4.54	5.23
**2**	4.61	5.37	4.79	3.01	5.02	4.62	4.92	5.09
**3**	5.06	5.97	5.40	3.39	5.64	5.26	5.35	5.86
**4**	4.87	5.26	5.98	3.39	5.29	4.73	4.77	5.51
**5**	4.93	4.66	4.58	3.17	4.72	4.48	4.54	5.13
**6**	4.64	5.37	4.79	3.40	5.02	4.62	4.92	5.03
**7**	5.06	5.97	5.40	3.78	5.64	5.26	5.35	5.80
**8**	4.91	5.26	5.98	3.78	5.29	4.73	4.77	5.48
**9**	4.92	4.66	5.58	3.17	4.72	4.48	4.41	5.13
**10**	4.64	5.37	4.79	3.40	5.02	4.64	4.92	5.03
**11**	4.93	4.66	4.58	3.17	4.72	4.48	4.87	5.13
**12**	4.59	5.37	4.79	3.40	5.02	4.62	4.92	5.03
**13**	5.04	5.97	5.40	3.78	5.64	5.26	5.35	5.80
**14**	5.87	5.53	5.30	4.24	5.88	5.46	5.63	6.12
**15**	5.54	6.24	5.51	4.47	6.19	5.60	5.80	6.12
**16**	6.10	6.84	6.13	4.83	6.80	6.24	6.28	6.90
**17**	5.63	6.13	6.70	4.83	6.45	5.72	5.98	6.54
**18**	5.85	5.53	5.30	4.24	5.88	5.46	5.63	6.11
**19**	5.54	6.24	5.51	4.47	6.19	5.60	5.80	6.12
**20**	6.05	6.84	6.13	4.83	6.80	6.24	6.28	6.90
**21**	5.60	6.13	6.70	4.83	6.45	5.72	5.98	6.54

**Table 3:** The R_M0_ values, b (slope), r (correlation coefficient), and LogP_TLC_ of the equation. R_M_ = R_M0_ + b·C for compounds **1**–**21**.

No. of Compound	R_M0_	r	b	LogP_TLC_
**1**	3.15	0.9953	−0.0383	4.26
**2**	4.14	0.9954	−0.0503	5.57
**3**	4.30	0.9944	−0.058	5.77
**4**	3.35	0.9953	−0.0383	4.56
**5**	3.72	0.9927	−0.0459	5.03
**6**	4.24	0.9982	−0.0582	5.69
**7**	4.46	0.9988	−0.0534	5.97
**8**	3.96	0.9987	−0.0539	5.34
**9**	3.69	0.9969	−0.0498	4.99
**10**	4.37	0.9977	−0.0540	5.86
**11**	3.48	0.9960	−0.0462	4.72
**12**	4.21	0.9989	−0.0576	5.65
**13**	3.58	0.9928	−0.0478	4.85
**14**	4.24	0.9967	−0.0573	5.69
**15**	4.49	0.9965	−0.0527	6.01
**16**	4.60	0.9979	−0.0571	6.15
**17**	4.29	0.9985	−0.0529	5.76
**18**	3.81	0.9934	−0.0484	5.14
**19**	4.18	0.9931	−0.0538	5.58
**20**	4.34	0.9973	−0.0548	5.82
**21**	4.09	0.9977	−0.0507	5.50

**Table 4:** R_M0_ and logP_lit_ values, and b (slope) and r (correlation coefficient) of the equation R_M_ = R_M0_ + b·C for standards **I**–**V**.

Lipophilicity Parameter	Standard				
	I	II	III	IV	V
logP_lit_	1.21 [36]	1.87 [37]	3.18 [37]	4.45 [37]	6.38 [38]
R_M0_	0.78	1.16	2.51	3.33	4.69
-b	0.0162	0.0247	0.0328	0.0412	0.0564
r	0.992	0.994	0.997	0.998	0.998
logP_TLC_	1.21	1.70	3.43	4.49	6.24

**Table 5:** Predicted parameters of the ADME and drug safety profiling for compounds **1**–**21**.

No. of Compound	Caco-2 [10^−6^ cm/s]	PPB [%]	CNS	HIA [%]	P-gp Substrates	CYP1A2 Inhibitor	CYP2C9 Inhibitor	CYP2C19 Inhibitor	CYP2D6 Inhibitor	CYP3A4 Inhibitor
**1**	226	99	−3.30	100	0.30	0.61	0.34	0.49	0.48	0.53
**2**	231	99	−3.23	100	0.38	0.57	0.26	0.49	0.46	0.50
**3**	207	100	−3.44	100	0.35	0.62	0.33	0.52	0.48	0.53
**4**	235	100	−3.96	100	0.35	0.68	0.40	0.51	0.42	0.54
**5**	226	99	−3.30	100	0.30	0.58	0.34	0.50	0.48	0.58
**6**	231	99	−3.23	100	0.38	0.61	0.26	0.49	0.46	0.54
**7**	207	100	−3.44	100	0.35	0.55	0.33	0.51	0.48	0.53
**8**	235	100	−3.96	100	0.35	0.67	0.40	0.50	0.37	0.54
**9**	230	99	−3.30	100	0.30	0.58	0.34	0.50	0.48	0.58
**10**	231	99	−3.23	100	0.38	0.61	0.26	0.49	0.46	0.54
**11**	211	99	−3.30	100	0.30	0.58	0.34	0.50	0.48	0.58
**12**	231	99	−3.23	100	0.38	0.61	0.26	0.49	0.46	0.54
**13**	207	100	−3.44	100	0.35	0.55	0.33	0.51	0.48	0.53
**14**	131	100	−3.51	100	0.28	0.66	0.33	0.51	0.49	0.53
**15**	163	100	−3.40	100	0.38	0.68	0.26	0.53	0.47	0.50
**16**	94	100	−3.72	100	0.38	0.63	0.32	0.57	0.50	0.51
**17**	137	100	−4.30	100	0.34	0.71	0.39	0.53	0.43	0.56
**18**	131	100	−3.51	100	0.28	0.66	0.33	0.51	0.49	0.53
**19**	163	100	−3.40	100	0.38	0.68	0.26	0.53	0.47	0.50
**20**	94	100	−3.72	100	0.38	0.63	0.32	0.57	0.50	0.51
**21**	137	100	−4.30	100	0.34	0.71	0.39	0.53	0.43	0.56

## Data Availability

Data from the research described in the manuscript are available from the authors.

## Supplementary Materials

The following supporting information can be downloaded at: https://www.mdpi.com/article/10.3390/molecules29071683/s1, Table S1. Obtained linear correlations between theoretical and chromatographic parameters of lipophilicity of the tested compounds **1**–**21**. Table S2. The molecular descriptors for compounds **1**–**21**. Table S3. The correlation of the logP_TLC_ values with the molecular descriptors and predicted ADME activities for compounds **1**–**21**. Table S4. The parameters of Lipinski’s, Ghose’s, Veber’s and Egan’s rules for compounds **1**–**21**.
