# Supplementary material for: Evaluation of the Lipophilicity of Angularly Condensed Diquino- and Quinonaphthothiazines as Potential Candidates for New Drugs
Source: Molecules. 2024 Apr 8;29(7):1683. doi: 10.3390/molecules29071683 (PMC11013424; doi:10.3390/molecules29071683)
Supplement: Supplementary file 1 [file molecules-29-01683-s001.zip › molecules-2903152-supplementary.pdf]

Evaluation of the Lipophilicity of Angularly Condensed Diquino- and Quinonaphthothiazines as Potential Candidates for New Drugs

Daria Klimoszek [1], Małgorzata Jeleń [2,*], Beata Morak-Młodawska [2] and Małgorzata Dołowy [3]

[1] Faculty of Pharmaceutical Sciences in Sosnowiec, Doctoral School, Medical University of Silesia in Katowice, Poland; d201204@365.sum.edu.pl (D.K.)
[2] Department of Organic Chemistry, Faculty of Pharmaceutical Sciences in Sosnowiec, Medical University of Silesia in Katowice, Jagiellońska 4, 41-200 Sosnowiec, Poland; bmlodawska@sum.edu.pl (B.M.M.)
[3] Department of Analytical Chemistry, Faculty of Pharmaceutical Sciences in Sosnowiec, Medical University of Silesia in Katowice, 41-200 Sosnowiec, Poland; mdolowy@sum.edu.pl (M.D.)
* Correspondence: manowak@sum.edu.pl

Content:

1. Table S1. Obtained linear correlations between theoretical and chromatographic parameters of lipophilicity of the tested compounds **1–21**. 2
2. Table S2. The molecular descriptors for compounds **1–21**. 2
3. Table S3. The correlation of the $logP_{TLC}$ values with the molecular descriptors and predicted ADME activities for compounds **1–21**. 3
4. Table S4. The parameters of Lipinski's, Ghose's, Veber's and Egan's rules for compounds **1–21**. 3

Table S1. Obtained linear correlations between theoretical and chromatographic parameters of lipophilicity of the tested compounds **1–21**. (r - correlation coefficient; s - standard error of estimation; p - significance level; F- ratio).

Compounds	Lipophilicity parameter	Equation	r	p-value	s	F-ratio
**1-21**	AClogP	AClogP = 0.8996logP_{TLC} + 0.7407	0.670	0.000	0.488	18.22
**1-21**	MLOGP	MLOGP = 0.7383logP_{TLC} – 0.1778	0.563	0.008	0.574	8.84
**1-21**	XLOGP2	XLOGP2 = 0.7824logP_{TLC} + 1.3324	0.578	0.006	0.586	9.53
**1-21**	XLOGP3	XLOGP3 = 0.6477logP_{TLC} + 1.6152	0.570	0.007	0.495	9.16
**1-21**	LogP	LogP = 0.6969logP_{TLC} + 1.5062	0.616	0.003	0.472	11.64
**1-21**	ClogP	ClogP = 0.5503logP_{TLC} + 2.7581	0.450	0.041	0.579	4.83

Table S2. The molecular descriptors for compounds **1–21**.

Compounds	Molar mass (M)	Molar volume (V_M)	Molar refractivity (Ref_M)
**1**	301.37	254.81	87.58
**2**	315.39	271.91	92.58
**3**	341.43	299.73	102.03
**4**	339.11	294.25	100.16
**5**	301.37	254.81	87.58
**6**	315.39	271.75	92.58
**7**	341.43	299.73	102.03
**8**	339.11	294.25	100.16
**9**	301.37	254.81	87.58
**10**	315.39	271.75	92.58
**11**	301.37	254.81	87.58
**12**	315.39	271.75	92.58
**13**	341.43	299.73	102.03
**14**	300.38	258.97	90.06
**15**	314.40	275.91	95.06
**16**	340.44	303.88	104.51
**17**	338.43	298.41	102.65
**18**	300.38	258.97	90.06
**19**	314.40	275.91	95.06
**20**	340.44	303.88	104.51
**21**	338.43	298.41	102.65

Table S3. The correlation of the $logP_{TLC}$ values with the molecular descriptors and predicted ADME activities for compounds **1–21**. (r - correlation coefficient; s - standard error of estimation; p - significance level; F- ratio).

Compounds	Molecular Descriptor and ADME Activities	Equation	r	p-value	s	F-ratio
**1-21**	Molar volume	$V_M = 15.62 logP_{TLC} + 194.72$	0.428	0.053	17.481	4.27
**1-21**	Molar refractivity	$Ref_M = 5.3645 logP_{TLC} + 66.692$	0.445	0.043	5.734	4.68
**1-21**	Caco-2	$Caco\text{-}2 = -42.984 logP_{TLC} + 421.64$	0.451	0.040	45.196	4.84
**1-21**	P-gp	$P\text{-}gp = 0.0411 logP_{TLC} + 0.1211$	0.584	0.005	0.030	9.86
**1-21**	CYP2C9	$CYP2C9 = -0.0387 logP_{TLC} + 0.5331$	0.417	0.062	0.045	3.99
**1-21**	CYP2C19	$CYP2C19 = 0.0209 logP_{TLC} + 0.4000$	0.459	0.036	0.021	5.08
**1-21**	CYP3A4	$CYP3A4 = -0.0212 logP_{TLC} + 0.6513$	0.439	0.046	0.023	4.54

Table S4. The parameters of Lipinski's. Ghose's. Veber's and Egan's rules for compounds **1–21**.

Compounds	H-Bond Acceptors	H-Bond Donors	Rotatable Bonds	TPSA [Å^2]	Lipinski's Rules	Ghose's Rules	Veber's Rules	Egan's Rules
**1**	2	1	0	63.11	+	+	+	+
**2**	2	0	0	54.32	+	+	+	+
**3**	2	0	2	54.32	+	+	+	+
**4**	2	0	1	54.32	+	+	+	+
**5**	2	1	0	63.11	+	+	+	+
**6**	2	0	0	54.32	+	+	+	+
**7**	2	0	2	54.32	+	+	+	+
**8**	2	0	1	54.32	+	+	+	+
**9**	2	1	0	63.11	+	+	+	+
**10**	2	1	0	63.11	+	+	+	+
**11**	2	1	0	63.11	+	+	+	+
**12**	2	0	0	54.32	+	+	+	+
**13**	2	0	0	54.32	+	+	+	+
**14**	1	1	0	50.22	+	+	+	+
**15**	1	0	0	41.43	+	+	+	+
**16**	1	0	2	41.43	+	-	+	+
**17**	1	0	1	41.43	+	+	+	+
**18**	1	1	0	50.22	+	+	+	+
**19**	1	0	0	41.43	+	+	+	+
**20**	1	0	2	41.43	+	-	+	+
**21**	1	0	1	41.43	+	+	+	+
